# Radioprotection for Astronauts’ Missions: Numerical Results on the Nomex Shielding Effectiveness

**DOI:** 10.3390/life13030790

**Published:** 2023-03-15

**Authors:** Filomena Loffredo, Emanuele Vardaci, Davide Bianco, Antonio Di Nitto, Maria Quarto

**Affiliations:** 1 Advanced Biomedical Sciences Department, University of Naples Federico II, 80131 Naples, Italy; 2 National Institute for Nuclear Physics (INFN), 80126 Napoli, Italy; 3 Department of Physics “E. Pancini”, University of Naples Federico II, Corso Umberto I, 80138 Naples, Italy; 4 Italian Aerospace Research Centre (CIRA), Via Maiorise, 81043 Capua, Italy

**Keywords:** radiation protection, shield, space radiation, galactic cosmic radiation (GCR), Nomex, monte carlo, Geant4

## Abstract

Space missions with humans expose the crews to ionizing radiation, mainly due to the galactic cosmic radiation (GCR). All radiation protection programs in space aim to minimize crews’ exposure to radiation. The radiation protection of astronauts can be achieved through the use of shields. The shields could serve as a suit to reduce GCR exposure and, in an emergency, as a radiation shelter to perform necessary interventions outside the space habitat in case of a solar proton event (SPE). A space radiation shielding that is suitable for exploration during space missions requires particular features and a proper knowledge of the radiation type. This study shows the results of numerical simulations performed with the Geant4 toolkit-based code DOSE. Calculations to evaluate the performance of Nomex, an aramidic fiber with high mechanical resistance, in terms of dose reduction to crews, were performed considering the interaction between protons with an energy spectrum ranging from 50 to 1100 MeV and a target slab of 20 g/cm^2^. This paper shows the properties of secondary products obtained as a result of the interaction between space radiation and a Nomex target and the properties of the secondary particles that come out the shield. The results of this study show that Nomex can be considered a good shield candidate material in terms of dose reductions. We also note that the secondary particles that provide the greatest contribution to the dose are protons, neutrons and, in a very small percentage, α-particles and Li ions.

## 1. Introduction

It is widely known that astronauts in space missions are exposed to various health risks, with consequent problems such as the psychological problems caused by isolation, medical problems caused by microgravity and acute and late effects caused by exposure to radiation [1,2,3,4].

The risk associated with exposure to radiation in the space environment includes both deterministic and stochastic effects. The component of space radiation to which an individual is exposed and for how long determines the type of risk [5].

Generally, intense solar particle events (SPEs), if not adequately shielded, are associated with acute effects. Instead, chronic exposure to galactic cosmic radiation (GCR) is associated with later effects, including cancer and other diseases of old age [6,7,8,9]. This radiation type is very different from the natural radiation to which we are exposed on Earth. On Earth, three countermeasures can be considered to limit radiation exposure (change in exposure time, distance between source and target, and use of shielding), while, in space, shielding is the only possible countermeasure that can be employed.

In this regard, astronauts are exposed to considerably higher radiation doses during missions than the limits imposed on both the population on Earth and professionally exposed workers, event though the latter, in practice, receive lower average doses than the annual limits. Therefore, astronauts are subject to a greater risk of exposure to radiation and, for this reason, the risk coefficients, especially in relation to age and gender, must be carefully examined. Radiation protection for astronauts is, therefore, of paramount importance. In a radioprotection program, the spacesuit, spacecraft and storm shelter should be designed to ensure to afford that the astronaut is as protected as possible. In fact, the radiation exposure should be kept as low as reasonably achievable (ALARA). Exposure limits, in terms of effective doses for astronauts engaged in missions in relation to gender and age, are based on the 3% risk of mortality from radiation-induced cancer [10,11]. In the space environment, radiation protection is a very complex problem because the radiation field is mixed. However, cosmic radiation also interacts with the shields and produces secondary radiation, whose dangerousness must be also evaluated. Products escaping from the shield and interacting with the astronauts could be either more or less harmful than the primary radiation. In this regard, the risk assessment and reduction in radiation exposure cannot be disconnected from the chemical composition of the shield and depends on the nature of the incident flux. To provide an estimation of such dependencies, it is necessary to know the type and energy spectrum constituting the primary radiation, build a tool to predict the nuclear reactions that occur during the interaction between the radiation with the shield and, finally, to transport the secondary radiation through the shield. The final goal is to estimate the dose the astronauts received due to the radiation that punched through the shield. The development of such a tool requires an in-depth knowledge of the chemical composition of the shield, the type and energy spectrum of the radiation fields, and a set of models used to treat nuclear reactions in the potentially large energy spectrum of the incident radiation field.

The space radiation field is usually divided into three categories: (i) Van Allen radiation belts, which consist of charged particles, mainly electrons and protons, which are trapped in the magnetic field of the Earth due to the Lorentz force; (ii) galactic cosmic radiation, composed mainly of protons, α particles (low percentage) and a very small percentage of heavy ions; (iii) solar particle event (SPE), a violent flow of charged particles composed by protons, helium and heavy ions [12,13].

The particle fluxes of the GCR and the SPE differ mainly in their dependence on solar activity and their energy spectra. In particular, an increase in solar activity produces an increasing frequency of SPE events and a decreasing GCR background [14]. The most dominant radiation component of the space field is constituted by the GCRs with an energy range up to 10^20^ eV. In this study, the energy spectrum obtained by the SPENVIS software ranged from 50 MeV to 1100 MeV. The GCR CREME96 model [15] implemented in SPENVIS is based on the Nymmik model. This is a semiempirical model and describes the flux variations in the particles that make up the GCR by defining certain parameters [16,17,18]. The energy spectra of GCRs can be sampled with a Monte Carlo method and, using transport codes such as Geant4, it is possible to implement a code for the nuclear interactions between such GCRs and the shield, and the transport the reaction produces using this shield. Geant4 is a tool with which it is possible to study the production and transport of radiation through matter.

This work follows a series of works on this topic, which were carried out considering several shield materials and the protons as the most abundant component of GCRs (about 87%). As anticipated in the previous work, reported in [18], the study conducted in this paper is its natural continuation. In the DOSE code developed based on the Geant4 tool, the electromagnetic and hadronic physical models of the interactions were implemented, as described in detail in previous works [19,20]. Here, we show the results of extending such works to the proton–shield interaction by considering the whole proton range, instead of few energies only (50, 100, 200, 500, 800 and ~1000 MeV), as in [18], to evaluate the properties of the secondary radiation produced in the target by the interactions between a beam of protons in an energy range that characterizes the GCR spectrum. The aim of the present work is to provide an estimate of the dose established due to the secondary radiation produced in the shield, during the whole proton energy range, and when exiting the shield. The dose was computed by considering the linear energy transfer (LET) of the radiation exiting the shield and hitting an ionization chamber of a given size. This scheme was chosen because it constitutes a typical geometry and detection setup whose experimental data can be compared to model calculations.

The choice of the proper material for shielding, in addition to its mechanical characteristic features, also pertains to its ability to minimize the escape of secondary radiation with the aim of reducing the dose absorbed by the astronauts behind the shield. As in Ref. [18], the present study was performed using the Nomex material as a shield and considering its molecular constitution. This is a heat- and flame-resistant meta-aramid fiber used in a wide range of applications, especially fabrics used to make protective clothing. In particular, the alveolar Nomex is also widely used for numerous engineering and scientific applications in the aerospace, construction, military, marine, and other sectors thanks to its properties of mechanical resistance, lightness and durability, which make it a very versatile material [21].

## 2. Materials and Methods

### 2.1. GCR

**Figure 1:**
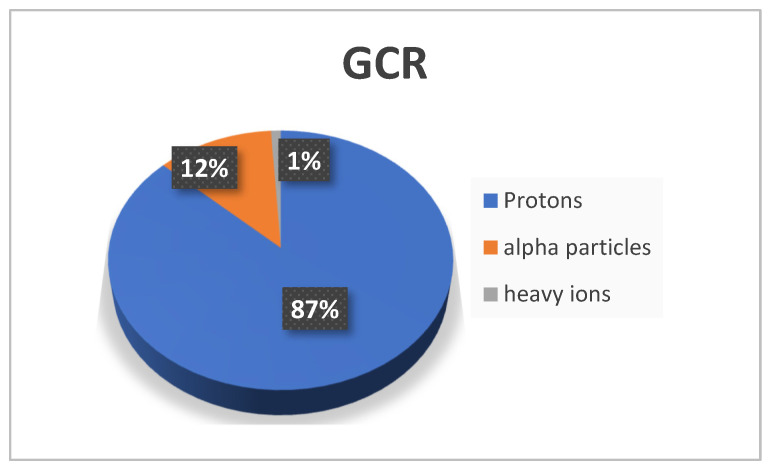
The galactic cosmic rays’ composition.

Space radiation consists of many kinds of energetic particles of different origins. Of the three radiation sources, which are described in the previous section, the dominant one is the GCR, which consists of particles with a charge ranging from Z = 1 (hydrogen) to Z = 92 (uranium), arriving from outside the heliosphere [22,23]. These particles are isotropically distributed. Due to their high energy (up to 10^20^ eV), it has been hypothesized that they originate from highly energetic phenomena such as supernova explosions or neutron stars [24]. GCR is composed of 98% of baryons and 2% electrons. The baryonic component consists of 87% protons and 12% alpha particles, with a small number of heavier nuclei, ~1%, as shown in Figure 1.

The GCR contributes more than 80% of the effective dose for crews involved in missions on the International Space Station (ISS). The National Council on Radiation Protection and Measurements (NCRP) recommended evaluating the equivalent dose by considering the quality factor as a continuous function of the linear energy transfer (LET) due to the complexity of the radiation space spectrum [25,26]. In fact, it is composed of a mixture of charged particles of the primary and secondary beams, which are characterized by a wide range of energies. The GCR flux is highly dependent on solar activity and is not constant. During the high-solar-activity phases, the fluxes in cosmic rays decrease compared to the minimum solar activity phases [27,28,29,30].

### 2.2. Radiation Shielding

First, a passive shielding for astronaut space exploration must be light, because very heavy shields are neither practical to wear nor practical to carry on spaceships. Second, it should minimize the production and transport of secondary radiation produced by the interaction between primary radiation and materials, which can be more harmful to astronauts’ health [31]. One of the shield materials considered in [18] is the Nomex, an aramidic fiber composed of C (54%) H (4%) N (9%) O (10%) Cl (23%), in addition to a certain percentage of air contained in the honeycomb structure. Nomex is characterized by a high mechanical resistance, which is a fundamental requirement for use in space [32].

### 2.3. Radiation–Matter Interaction

For radiation protection purposes, the study of the interactions of cosmic radiation as it passes through materials of different natures and thicknesses aims to optimize shielding. Charged particles, when interacting with target atoms, lose energy in the absorber material through the following fundamental processes: (i) ionization and excitation (inelastic collisions); (ii) nuclear reactions that lead to fragmentation of the particles involved in collisions with the production of lighter fragments. Inelastic collisions represent the main contributor of energy loss in matter. The energy is transferred to atoms causing an excitation (“soft” collisions) or ionization with the consequent expulsion of the electron (“hard” collisions). Generally, a small amount of energy is transferred in each collision. Instead, elastic collisions when charged particles interact with target nuclei are less frequent. Furthermore, the nucleus of most materials is much heavier than the charged particles (such as the protons treated in the specific case); therefore, very little energy is transferred in the collision.

### 2.4. Monte Carlo Simulation—Geant4

Geant4 is an open-source tool that was initially designed for high-energy experiments, and is also applied outside this field, i.e., in the medical, biological, space and radioprotection fields. With this instrument, it is possible to simulate the production and transport of radiations through matter using the Monte Carlo method. Geant4 provides many different useful functions to simulate radiation–matter interactions: (i) it allows for a geometry and graphical display of the experimental setup, materials, and radiation source to be built; (ii) the physical processes can be implemented; (iii) the motion of the particles in the material can be tracked and viewed; (iiii) the response of sensitive components of a detector can be recorded [33,34,35].

In Geant4, each interaction process is described by multiple models, each valid for a specific energy range, ranging from a few eV to TeV, and is characterized by its own cross-section. The latter depends on the energy of the incident particle and the characteristics of the medium that is crossed. These are obtained through theoretical models or on the basis of experimental data libraries implemented in the toolkit. If the latter are absent or insufficient, they are obtained through parameterizations.

All physical processes in the Geant4 toolkit, involve two distinct phases: (i) calculation and use of the total cross-section; (ii) the generation of the final state. These processes, depending on their physical nature, are divided into two main categories: electromagnetic and hadronic interactions.

As the core of the DOSE code is the study of the interactions of the matter of a proton beam of a wide range of energies, the main physical processes are elastic and inelastic collisions where the energy loss in each collision is a very small percentage of the initial kinetic energy of the incident particle.

To evaluate the dose trends of a primary beam of 1 GeV protons before and after different material shields are used, and to validate the hadronic physical processes, a code named DOSE was developed. The comparison between the simulated dose and experimental measurements performed at the NASA Space Radiation Laboratory (NSRL) in Brookhaven, NY, USA [36] enabled the validation process of the code.

### 2.5. The Physical Models Implemented in Dose Code

In the following section, a short overview of the physical models implemented in DOSE code is given. The DOSE code was developed to evaluate physical processes belonging to the category of electromagnetic and hadronic interactions. As anticipated in the previous section, for the Geant4 tool, each interaction process is described by several models, with each depending on the type of particle and valid for a specific energy range. In particular, the physical models implemented in the DOSE code are as follows: (i) Penelope for e−, e+, γ; (ii) CHIPSElastic, Bertini and Binary for protons; (iii) HP Elastic, LElastic, CHIPSElastic, HP Inelastic Model, Bertini and Binary for neutrons; (iiii) binary reactions for ions. After a primary interaction, these models produce a cascade of new products, ranging from light to heavy particles. Such newly generated particles (secondary radiation) are transported through the material and can cause new interactions with a high enough energy. Some can definitively escape the material. The cascade process originating from the primary interaction terminates when the primary and/or secondary radiation exit the shield.

### 2.6. Geometry Used in the Calculations for the Dose Evaluation

**Figure 2:**
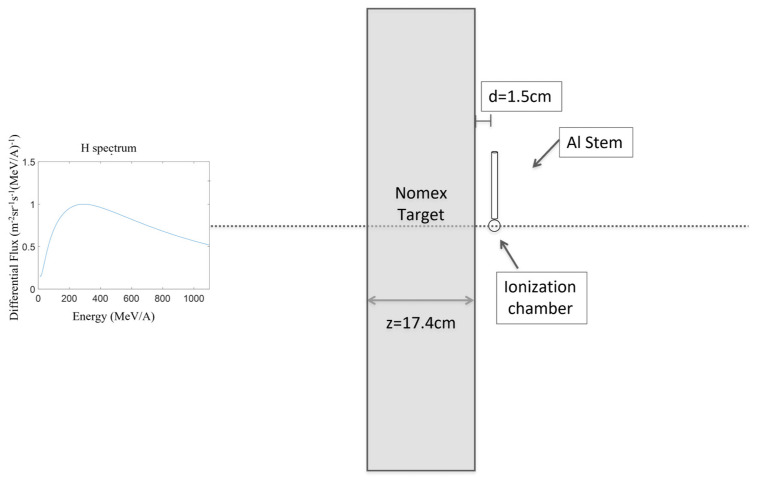
A schematic view of the geometry of the experimental setup used in the simulation. (**left**) GCR spectrum; (**right**) a 20 g/cm^2^ thick Nomex target and the tissue-equivalent ionization chamber at 1.5 cm from the Nomex target [18].

Figure 2 shows a schematic drawing of the experimental setup implemented in the simulation. This consisted of the proton source with a GCR energy spectrum, the Nomex target, and the tissue-equivalent ionization chamber. The proton beam had a diameter of 20 cm and energy of up 1100 MeV in-air. The Nomex slab (see Table 1 for its physical properties) had a parallelepiped shape with a thickness of 20 g/cm^2^ (whereby z = 17.4 cm) in the direction of the primary beam. The slab had an area of 30 × 30 cm^2^ when facing the beam. The thickness used in the calculations is typical of the storm shelters used by the astronauts during intense SPEs [19,23]. The ionization chamber was positioned at a distance d = 1.5 cm behind the target to check the dose trend. For each emerging radiation that hits the ionization chamber, the event-by-event absorbed dose can be defined as the total energy carried by the radiation hitting the chamber. For the GCR spectrum energy, a run of 1.5 × 10^8^ events was simulated.

## 3. Results

### 3.1. Secondary Radiation Produced in the Target

**Figure 3:**
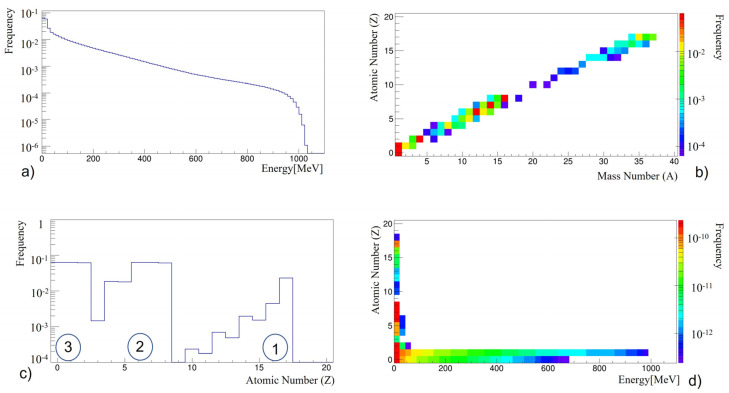
(**a**) Energy spectrum for the secondary products in the Nomex target normalized to the total yield; (**b**) computed atomic and mass numbers (Z, A) of the secondary products produced in proton–Nomex interaction for the GCR proton energy spectrum; (**c**) computed atomic numbers and event frequency of the secondary products produced in the proton–Nomex interaction for the GCR proton energy spectrum. The three clusters of high (1), middle (2), and low (3) masses are highlighted; (**d**) computed atomic number and energy (Z, E) of the secondary particles produced in the proton–Nomex interaction for the GCR proton energy spectrum [18].

Figure 3 shows the trends in the yield of secondary particles as a function of energy and atomic and mass numbers (Z and A, respectively) produced in the proton–Nomex interaction over the whole GCRs energy range. There is no substantial change in these plots compared to the ones presented in [18] and computed only for selected energy values. In this study, three main production clusters (Figure 3c) are shown to be located (1) at the larger masses around the mass of ^37^Cl, (2) around the masses of ^12^C and ^14^N, (3) in the correspondence of the light-particle masses with atomic number Z ≤ 2. The analysis of these three clusters provides the first piece of information: the protons interacting with the nuclei of C, N and Cl, using proton capture, produce composite systems that deexcite by emitting low-mass particles in a cascade. The other two clusters (2 and 3 in Figure 3c) are the consequence of a complex chain of nuclear reactions [18]. We would like to highlight the analogous production of neutrons. This is because neutron production occurs in all the relevant nuclear collisions (except the elastic ones). However, neutrons are more penetrating than charged particles and, for this reason, they could make an important contribution to the dose absorbed by astronauts. Therefore, when considering a shielding material, its ability to reduce the escape of neutrons [37,38] must be taken into account.

### 3.2. Transport of Primary and Secondary Radiation behind the Target

**Figure 4:**
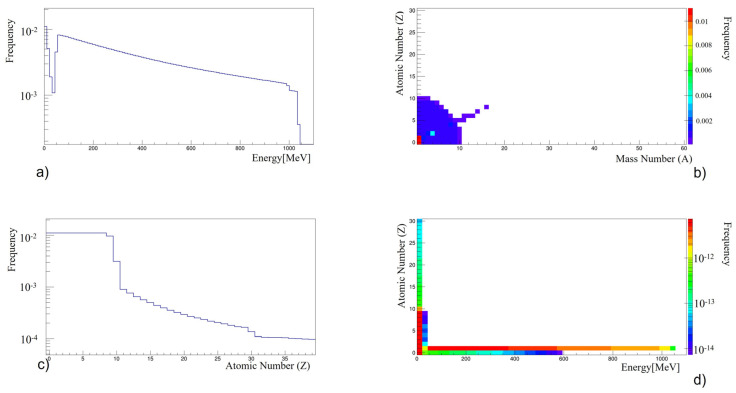
(**a**) Energy spectrum for the secondary products, normalized to the total yield that leaves the target and arrives at the ionization chamber; (**b**) computed atomic and mass numbers (Z, A) of the secondary particles that leave the target and arrive at the ionization chamber; (**c**) computed atomic numbers and event frequency of the secondary particles that leave the target and arrive at the ionization chamber; (**d**) computed atomic number and energy (Z, E) of the secondary particles that leave the target and arrive at the ionization chamber [18].

**Figure 5:**
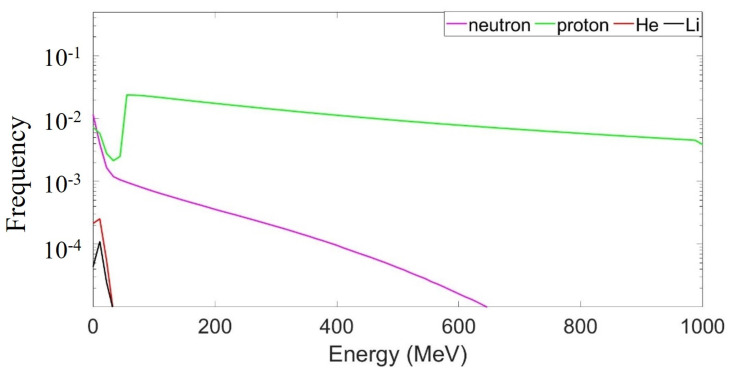
Energy spectra for the main secondary products that leave the target and arrive at the ionization chamber. Each spectrum is independently normalized to the total yield (y).

The radiation produced in the shield is then transported through the shield via the DOSE code. Part of it stops inside the shield itself because of the stopping power of the Nomex, while part of it appears outside the shield. After the target, a tissue-equivalent ionization chamber is placed 1.5 cm from the slab. Figure 4 shows the energy, Z and A spectra of the radiation coming out of and absorbed by the ionization chamber. The energy spectrum mostly comprises protons that are exiting almost undisturbed by the material. The drop in intensity to below 50 MeV (Figure 4a) is the consequence of the stopping power of the Nomex, which is 50 MeV for the whole target slab. Therefore, protons with an energy lower than 50 MeV are mainly stopped inside the material if they do not produce nuclear reactions. Table 2 summarizes the percentage of the secondary particles that impinge on the ionization chamber when leaving the shield. Heavier nuclei are stopped inside the Nomex material. Only the lightest and more energetic ions may escape. The energy spectrum of the particles in Table 2 is shown in Figure 5. Only protons and neutrons have an energy above 50 MeV and contribute heavily to the final dose. The different trends in each energy spectrum are due to the different nuclear processes involved. In order to be extended to energies above 50 MeV, the production of protons and neutrons must come from direct reaction channels such as stripping or knock-out reactions. In this case, neutrons or protons are produced in a narrow cone in the forward direction and move with almost the same velocity as the primary beam. This justifies the long tail of the neutron and proton spectra. Instead, protons, neutrons or alpha particles with a lower energy are mostly produced by compound nucleus reactions where the projectile fuses completely with the target nucleus. In this case, the energy spectrum is of a Maxwellian type due to the thermalization process of the compound nucleus before particle emission (the so-called process of “evaporation”). Furthermore, the emission is fairly isotropic, because the formation of the compound nucleus is independent of its decay and no memory of the initial beam direction is kept, as in the case of direct reactions. The absence of a long tail in the alpha particles and lithium ions means that they are both produced in compound nuclear reactions.

The plots in Figure 4 make it evident that the effect of the Nomex shield is not only to attenuate the proton intensity but also to produce other particles that could be potentially harmful. The overall dose could, therefore, be bigger with respect to the case without a shield due to this secondary radiation. In the next section, an evaluation of the dose is provided.

### 3.3. Estimate of the Dose

In the low-energy range, below 50 MeV, the largest dose amount is due to protons, neutrons and ions with a low Z (about Z ≤ 10), which have an intensity that is approximately one order of magnitude bigger than ions with 11 < Z < 20 (see Figure 4d). Considering the simulated radiation spectrum that releases its energy inside the ionization chamber, the overall effect is an increase in dose. In the position after the shield, for the Nomex materials, the increase is about 14%. The dose is normalized to that without a shield, using the same ionization chamber at −19 cm (on the left side in Figure 2). This increase in dose is expected. In Loffredo et al., 2019 [20] the best material in terms of absorbed dose at 1.5 cm from the shield was the PMMA, but this material is not suitable as a shield in space because it has a low mechanical resistance to traction. This work shows that the Nomex materials can guarantee a similar increase in dose to PMMA.

## 4. Summary and Conclusions

Radiation exposure is a major limit to human exploration during space missions. Passive shielding is the only available physical countermeasure at present. Studying the different shielding materials can lead to a reduction in dose for the astronauts. Therefore, attempts to increase the radiation protection of astronauts cannot disregard the study of particular shielding features that are suitable for the crew’s exploration during space missions, and a proper understanding of radiation physics. In this work, we analyzed the secondary products obtained following the interaction between a proton beam of a given spectrum and a slab of the Nomex, a possible candidate material to shield astronauts in space. The secondary radiation is the consequence of the elastic scattering and the nuclear reactions between the protons and the nuclei in the slab. Secondary radiation is transported in the same material and the dose that appears after this is computed by estimating the LET in a tissue-equivalent ionization chamber. The result is that Nomex produces an increase in dose of 14% compared to the case without a shield. This increase is similar to that observed for the PMMA material that is considered to be the best material for shielding, which is not suitable for spacecraft for mechanical reasons. From this study, we can conclude that Nomex could be considered as a good alternative candidate for both spacecraft shielding and astronaut radiation protection. In a future step, the authors intend to develop an extension of the present tool in which the energy spectra of all the particles that comprise the GCR can be included. This add-on is necessary because it is well known in the literature that both alpha particles (12%) and even very small percentages of heavy ions (about 1%) make an important contribution to the overall dose.

## Figures and Tables

**Table 1:** Information of the Nomex shield used in the simulation thickness, density and composition [18,20].

Materials Shield	Thickness (cm)	*ρ* (g/cm^3^)	Composition	Dose Increase
Nomex	17.4	1.15	[C(54%) H(4%) O(10%)N(9%) Cl(23%)]45%+ [N(70%) O(30%)]55%	14%

**Table 2:** Percentage of the secondaries that leave the shield and impinge on the ionization chamber.

Secondary	%
Protons	95
Neutrons	3
He	0.05
Li	0.02

## Data Availability

Supporting data includes data generated at the MC simulation. All of the data supporting the findings of the presented study are available from the corresponding author on request.
